# Proteomic mapping of atrial and ventricular heart tissue in patients with aortic valve stenosis

**DOI:** 10.1038/s41598-021-03907-3

**Published:** 2021-12-22

**Authors:** Boris Barbarics, Katja Eildermann, Lars Kaderali, Lukas Cyganek, Uwe Plessmann, Julius Bodemeyer, Thomas Paul, Philipp Ströbel, Henning Urlaub, Theodorus Tirilomis, Christof Lenz, Hanibal Bohnenberger

**Affiliations:** 1 Department for Pediatric Cardiology, Intensive Care and Pulmonology, University Medical Center, Robert-Koch-Str. 40, 37075 Göttingen, Germany; 2 DZHK (German Center for Cardiovascular Research), Partner Site of Göttingen, Göttingen, Germany; 3 Institute of Bioinformatics, University Medicine Greifswald, Felix-Hausdorff-Str. 8, 17475 Greifswald, Germany; 4 Department for Cardiology and Pneumology, University Medical Center, Robert-Koch-Str. 40, 37075 Göttingen, Germany; 5 Cluster of Excellence “Multiscale Bioimaging: From Molecular Machines to Networks of Excitable Cells” (MBExC), University of Göttingen, Göttingen, Germany; 6 Bioanalytical Mass Spectrometry Group, Max Planck Institute for Biophysical Chemistry, Am Fassberg 11, 37077 Göttingen, Germany; 7 Institute of Pathology, University Medical Center, Robert-Koch-Str. 40, 37075 Göttingen, Germany; 8 Institute of Clinical Chemistry, University Medical Center, Robert-Koch-Str. 40, 37075 Göttingen, Germany; 9 Department of Thoracic and Cardiovascular Surgery, University Medical Center, Robert-Koch-Str. 40, 37075 Göttingen, Germany

**Keywords:** Mass spectrometry, Proteomic analysis, Cardiac hypertrophy, Cardiology, Valvular disease, Pluripotent stem cells, Stem-cell differentiation

## Abstract

Aortic valve stenosis (AVS) is one of the most common valve diseases in the world. However, detailed biological understanding of the myocardial changes in AVS hearts on the proteome level is still lacking. Proteomic studies using high-resolution mass spectrometry of formalin-fixed and paraffin-embedded (FFPE) human myocardial tissue of AVS-patients are very rare due to methodical issues. To overcome these issues this study used high resolution mass spectrometry in combination with a stem cell-derived cardiac specific protein quantification-standard to profile the proteomes of 17 atrial and 29 left ventricular myocardial FFPE human myocardial tissue samples from AVS-patients. In our proteomic analysis we quantified a median of 1980 (range 1495–2281) proteins in every single sample and identified significant upregulation of 239 proteins in atrial and 54 proteins in ventricular myocardium. We compared the proteins with published data. Well studied proteins reflect disease-related changes in AVS, such as cardiac hypertrophy, development of fibrosis, impairment of mitochondria and downregulated blood supply. In summary, we provide both a workflow for quantitative proteomics of human FFPE heart tissue and a comprehensive proteomic resource for AVS induced changes in the human myocardium.

## Introduction

Aortic valve stenosis (AVS) is an important cause of mortality and morbidity and one of the most common valve diseases in the western world^1,2^. Progressive AVS causes increasing pressure overload and enhanced wall stress on the left ventricle by narrowing of the aortic valve opening^3^. The blood flow from the left ventricle into the aorta is obstructed and decreases even further when the left ventricle develops hypertrophy. When myocardial remodeling becomes maladaptive, AVS results in compensatory hypertrophy of the left ventricle, subendocardial ischemia and myocardial fibrosis^1,2^. To maintain efficient cardiac function the heart becomes hypertrophic by the addition of sarcomere units within cardiomyocytes (CMs)^3,4^. There is a strong relationship between the sarcomeric recycling ubiquitin–proteasome system, the autophagy system and cardiac disease, especially hypertrophy. Hypertrophic CMs can compress subendocardial arterioles leading to microvascular rarefaction and subendocardial ischemia^5^. Poor nourishment together with complex pathological signals cause mitochondrial dysfunction, metabolic reprogramming, and dysregulation of Ca^2+^ handling proteins, finally resulting in death of CMs. The replacement of dysfunctional CMs by myofibroblasts together with excessive extracellular matrix (ECM) production finally causes irreversible cardiac fibrosis^4^. The grade of preoperative myocardial fibrosis has emerged as the strongest independent predictor of mortality in AVS^6–8^.

When AVS patients develop the typical clinical symptoms, i.e., the triad of heart failure, angina, and syncope^3^ together with left ventricular dysfunction they are in urgent need for surgical or interventional aortic valve replacement (AVR). However, treatment may be delayed if symptoms are equivocal. Late AVR might prevent death but cannot preserve function. To improve patient care, a more detailed biological and molecular understanding of the changes that lead to myocardial decompensation and fibrosis, and biomarkers are needed for better risk stratification and to select patients that will benefit from urgent or earlier surgical treatment^3,9^.

In-depth analysis of the proteome of human heart tissue from affected patients offers a great opportunity to assess disease-related protein expression patterns^3,10^ as it was recently demonstrated by Schlotter et al*.* for calcific aortic valve disease^11^. Highly abundant proteins could be targets for medical treatment and proteome expression profiles could be measured in blood samples for diagnostic purposes.

During heart surgery myocardial tissue is routinely sampled, formalin-fixed and embedded in paraffin (FFPE) for diagnostic or surgery-related reasons. Specialized protocols enable protein isolation from FFPE tissue for mass spectrometric analysis. When suitable deparaffinization and de-crosslinking protocols are employed, mass spectrometric analysis of FFPE tissues can provide protein abundance profiles comparable to those obtained from fresh frozen tissues^12,13^. Still, robust longitudinal standardization strategies are required for accurate and robust quantification specifically where large sample cohorts are examined, or for e.g. longitudinal studies^14^. A metabolically heavy isotope-labeled protein quantification standard derived from cell culture can solve this problem^15^, however reliable cell culture models of human CMs have not been available. Correspondingly, accessible experimental protocols for protein quantification from FFPE human heart tissue have been critically lacking.

To overcome these methodical issues, we (1) aimed to establish a cell culture derived spike-in protein quantification standard labeled with heavy amino acids (SILAC = Stable Isotope Labeling by Amino acids in Cell culture) from induced pluripotent stem cells (iPSC) differentiated into atrial and ventricular CMs, allowing for accurate quantification of protein expression profiles. Recently, we were able to show that these cells, can be metabolically labeled for SILAC-based proteomics^16^. We then (2) present a reproducible workflow by combining this SILAC quantification standard with filter-aided sample preparation (FASP) of human FFPE heart tissue for global and unbiased high-resolution mass spectrometry of atrial and ventricular tissue from patients with classical severe AVS and concomitant septal hypertrophy^14^. Finally, we (3) selected potentially disease related proteins by comparing our results to published results from healthy human hearts^17^.

## Materials and methods

### Heart tissue samples

Heart tissue was obtained from 29 patients with classical AVS undergoing aortic valve replacement and septal, subvalvular left ventricular myocardial resection according to the Morrow technique^18,19^. Right atrial samples were taken from the right atrial appendage from the venous cannulation site^19^. Tissue was immediately fixed in 4.5% buffered formalin and embedded in paraffin. Patients with hypertrophic obstructive cardiomyopathy, bicuspid valve disease, low flow low gradient AVS, rheumatic disease or amyloidosis were excluded.

### Azan staining

3 µm thick FFPE tissue sections were deparaffinized following standard protocols and stained using the commercially available Azan (Heidenhain) staining kit from MORPHISTO^®^ (#12079) according to the manufacturer’s recommendations^20^. Azan staining visualizes collagenous and reticular connective tissue and acidic mucosubstances in blue, muscle tissue in orange to red and erythrocytes, nuclei, and glial fibrils in red.

### Generation of the protein quantification standard

Human iPSC-lines from two healthy donors were used in this study. Human iPSC lines UMGi001-A clone 1 (iWT.D2.1) and UMGi014-A clone 2 (isWT1.Bld2) were described previously^16^. These cell lines had been generated from dermal fibroblasts and peripheral blood mononuclear cells, respectively, using the STEMCCA lentivirus system or the integration-free CytoTune-iPS 2.0 Sendai Reprogramming Kit. Each of the two iPSC lines were differentiated into atrial and ventricular iPSC-CMs via modulation of WNT signaling and retinoic acid modulation and subsequent metabolic selection, as previously described^16^. Differentiated cultures were labeled with stable isotope-labeled arginine and lysine for 45 days in RPMI1640 (ThermoFisher), 2% B27 (ThermoFisher), 10 μl/ml Glutamax (ThermoFisher), 25 mM HEPES (ThermoFisher), 1.74 mM l-proline (Sigma-Aldrich), 0.219 mM ^13^C_6_,^15^N_2_-l-lysine and 0.575 mM ^13^C_6_,^15^N_4_-l-arginine (Cambridge Isotopes). SILAC-labeled cells were pelleted at day 65–69 of differentiation. Differentiation of iPSCs into atrial and ventricular CMs was performed in three separated replicates each, respectively. Proteins were isolated, protein concentrations determined, and equal amounts of protein mixed to generate the quantification standard to obtain equal representation of both atrial and ventricular proteins in the standard^16,21,22^.

### FFPE sample preparation for mass spectrometry

FFPE heart tissue samples were prepared for mass spectrometry analysis as described previously^21^. Briefly, areas containing at least 80% CMs were marked on an H&E-stained slide and corresponding areas were isolated by macrodissection. Cardiac samples were deparaffinized, dehydrated and lysed in 100 mM ammonium bicarbonate (ABC), pH 8.0, 4% sodium dodecyl sulfate, 0.2% deoxycholic acid and 50 mM Tris-(2-carboxyethyl)-phosphin. Protein concentrations were determined using the 660 nm Kit (ThermoFisher) following the manufacturer’s instructions. 50 µg of tissue lysates were mixed with equal amounts of protein quantification standard and 200 µl of 8 M urea and 0.2% Deoxycholic acid (DCA) in 100 mM ABC, pH 8.0 and loaded onto 30 kDa microcon filter (Millipore)^23^. All on-column washing steps were performed for 10 min at 14,000*g*. After 4 washing steps, proteins were alkylated with 50 mM Indole-3-acetic acid in 100 mM ABC, pH 8.0 for 60 min. Urea was then washed out with 0.2% DCA in 50 mM ABC, pH 8.0 and samples were digested using sequencing-grade trypsin (Promega) in a ratio of trypsin to protein of 1:50 (w:w) resulting in the use of 2 µg trypsin for 100 µg used protein. Peptides were collected using 50 mM ABC, pH 8.0 and mixed with 200 μl ethyl acetate and 2.5 µl trifluoroacetic acid (TFA). After sonification and centrifugation at 16000*g* the upper organic layer was removed, and samples were vacuum-dried and washed with 50% methanol. Resulting peptides were fractionated with Pierce™ High pH Reversed-Phase Peptide Fractionation Kit (Thermo scientific) and again vacuum-dried. Ostasiewicz et al. have shown that by using the FASP-protocol proteomic analysis of formalin fixed tissue are qualitatively and quantitatively equal to the analysis of fresh tissue^12^. A detailed protocol for “filter aided sample preparation” of formalin fixed tissue samples can be found in the “Supplementary Material and Methods”.

### Mass spectrometry analysis

Peptides were resuspended in sample loading buffer (2% acetonitrile and 0.05% TFA) and separated on an UltiMate 3000 RSLCnano HPLC system (ThermoFisher) hyphenated to a hybrid quadrupole-orbitrap mass spectrometer Q Exactive HF-X (ThermoFisher). First, the peptides were desalted on a reverse-phase C18 pre-column (Dionex, 5 mm × 0.3 mm ID) for 3 min. Subsequently the pre-column was switched in line with the analytical column (300 mm × 0.075 mm ID) packed in-house with ReproSil-Pur C18 AQ 1.9 μm reversed-phase resin (Dr. Maisch GmbH). Solvent A consisted of 0.1% formic acid in water, and solvent B consisted of 80% acetonitrile and 0.1% formic acid in water. Peptides were eluted using a linear gradient of 5–42% B over 166 min at a flow rate of 300 nL/min. The temperature of the pre-column and the column was maintained at 50 °C. The mass spectrometer was operated in Top30 data-dependent acquisition mode, where the most intense 30 precursors within the *m/z* range of 350–1500 were selected for MS2 fragmentation with a Normalized Collision Energy of 28%. MS2 spectra were acquired in the Orbitrap at a resolution setting of 15,000 FWHM (Full Width Half Maximum), a maximum ion Trap Fill Time of 60 ms and an AGC target of 1.0e^5^, respectively. Dynamic Exclusion was set to 45 s.

MS/MS spectra were searched against a UniProtKB/Swiss-Prot human database containing 134,921 protein entries supplemented with 245 frequently observed contaminants collated with the Andromeda search engine^24^. Precursor and fragment-ion mass tolerances were set to 6 and 20 ppm, respectively, after initial recalibration. Protein N-terminal acetylation and methionine oxidation were allowed as variable modifications. Cysteine carbamidomethylation was defined as a fixed modification. Minimum peptide length was set to seven amino acids, with a maximum of two missed cleavages. The false discovery rate was set to 1% at both the peptide and the protein level by using a forward-and-reverse concatenated decoy database approach. For SILAC quantification, multiplicity was set to two-channel (Lys + 0/Arg + 0, Lys + 8/Arg + 10) labeling. At least two ratio counts were required for peptide quantification, and the “re-quantify” option was enabled. All raw files and MaxQuant search results have been deposited in the ProteomeXchange Consortium via the PRIDE partner repository with the dataset identifier PXD014297^25^.

### Statistical analysis

Statistical analysis was performed in R version 3.5.3 (R-Core-Team, 2019) and in Bioconductor version 3.4 to obtain candidate proteins that were differentially expressed between the atrial and ventricular samples^26,27^. In brief, peptides with more than 2/3 missing values were excluded from further analysis. For the remaining proteins, missing values were imputed by using k-nearest-neighbor imputation with k = 10. Univariate analysis was performed with the Bioconductor package limma, using the lmFit and eBayes functions. P-values were corrected by using the false discovery rate^28,29^.

### Immunhistological stainings

For immunhistological stainings 2 µm paraffin sections were deparaffinized and rehydrated by incubation in xylol and a series of descending ethanol concentrations. After blocking of endogenous peroxidase and incubation in Target Retrieval Solution either with low or high pH (Dako/Agilent), the tissue was incubated over night with antibodies against NPPA (1:5000, ph low, rabbit, Sigma/Atlas HPA #058269) or against MLC2a (1:400, pH high, mouse, Synaptic Systems #311011) at 4 °C. Then a suitable secondary antibody (Zytochem Plus HRP Polymer anti-Rabbit ZUC032-006 or Rabbit Anti Mouse DAKO PO260) was applied followed by DAB reaction. All stained sections were covered and scanned using the Dotslide system (Olympus).

### Overrepresentation analysis

Significantly upregulated proteins of atrial and left ventricular myocardium were used to perform the PANTHER Overrepresentation test (released 2020-08-10) provided on http://www.geneontology.org. As a result, we obtained a list of significantly enriched cellular component/related gene ontology terms for right atria and left ventricles.

### Protein–protein comparison

To compare our dataset to data from healthy hearts, all proteins from differential protein expression analysis were correlated with the respective list of atria/ventricles differential expression analysis published by Doll et al*.* (file: 41467_2017_1747_MOESM7)^17^ in Microsoft Excel. Respective fold change and adjusted p-values were noted. Linear regressions of the fold change values between the two datasets were calculated using GraphPad Prism version 9 using Deming linear regression. Proteins with a Y-distance from the regression line greater than 2 and a significant fold change in at least one of the studies were defined as potentially disease-relevant.

### Ethical approval

This study was approved by the Ethical Committee of Göttingen University Medical Center (#10/9/15). All procedures performed in studies involving human participants were in accordance with the ethical standards of the institutional and/or national research committee and with the 1964 Helsinki declaration and its later amendments or comparable ethical standards.

### Informed consent

Informed consent was obtained from all individual participants included in the study.

## Results

### Patient cohort

For proteomic analysis, we collected the septal portion of the left ventricle from 29 patients during Morrow myectomy^18,19^. Due to procedural limitations in the operating theater, it was only possible to obtain 17 atrial samples simultaneously from all included 29 patients. Preoperative patient characteristics are summarized in Table 1.

**Table 1:** Patient characteristics with aortic valve stenosis.

	Left ventricle sample (n = 29)	Right atrium sample (n = 17)
Age: mean [years] (range)	70 (51–84)	69 (53–84)
Men/women	15/14	5/12
Ejection fraction [%]: mean (range)	56 (35–80)	61 (35–80)
Preoperative NYHA class	II–III	II–III
Mean AV pressure gradient [mmHg]	41	36
Mean maximum velocity [m/s] (range)	4 (2–4.7)	3,8 (2.6–4.7)
Mean septal hypertrophy measures [mm]	15 (9–26)	15
Mean AV area [cm^2^] (range)	0.8 (0.3–1)	0.7 (0.3–1)
Aortic valve insufficiency	22	13
CABG or stent	18	10
Atrial fibrillation [n]	10	8
Arterial hypertension [n]	28	15

*AV* aortic valve, *CABG* coronary artery bypass graft, *NYHA* New York Heart Association.

### Tissue histology

**Figure 1:**
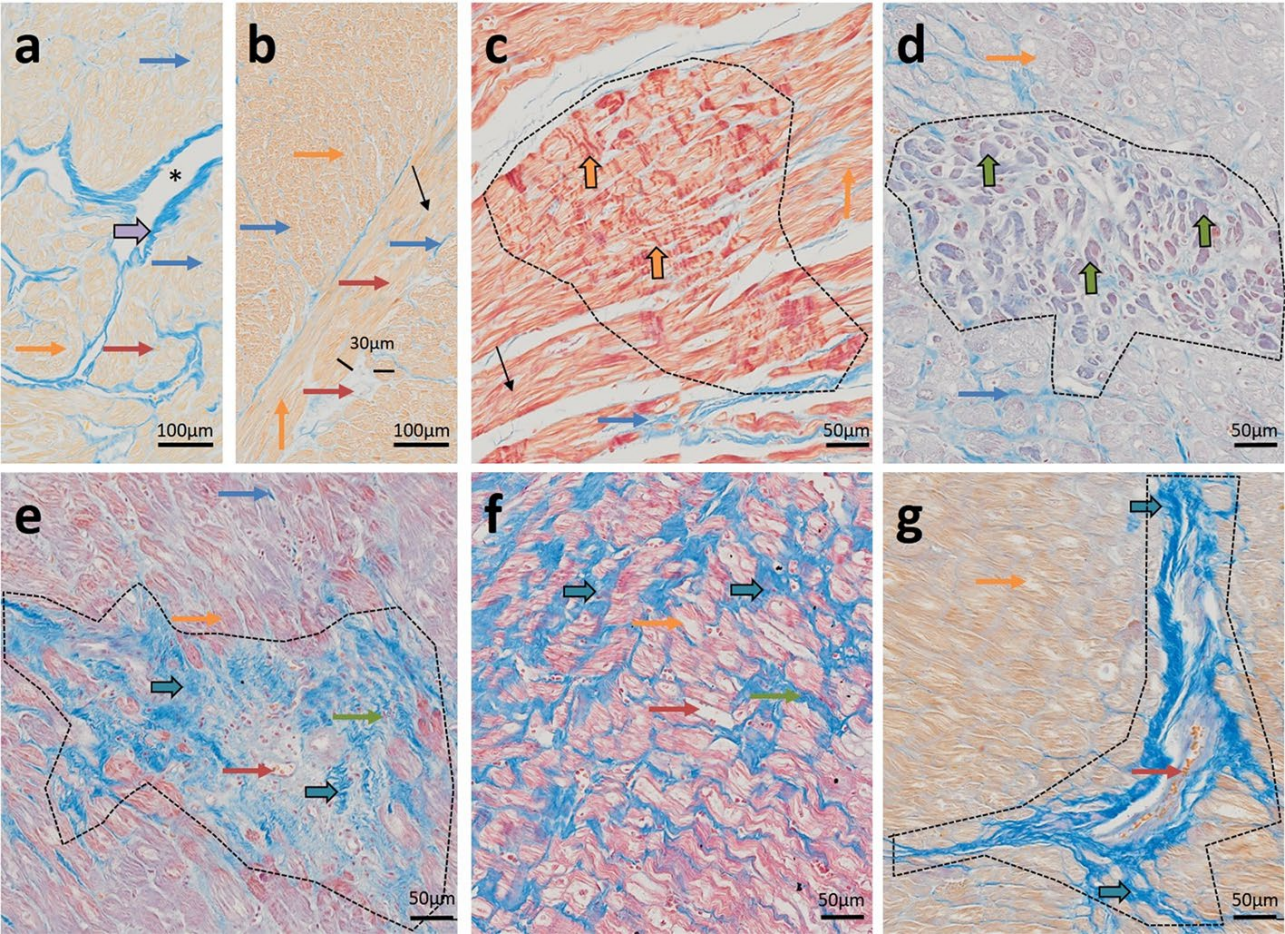
Azan staining of atrial and diseased ventricular tissue. Cardiomyocytes (CMs) are stained orange to pink; connective tissue is visualized in blue. (**a**) Atrium with endocardial tissue surrounding islets of myocardial tissue. Myocardial tissue is interspersed with interstitial tissue. (**b**) Hypertrophic myocardium containing enlarged CMs (cell diameter approx. 35 µm) and scant interstitial tissue without additional signs for decompensation. (**c**) Severe contraction banding. (**d**) Atrophic CMs. (**e**) Replacement fibrosis. (**f**) Interstitial fibrosis. (**g**) Perivascular fibrosis. Thin blue arrow: interstitial tissue, thin orange arrow: CMs (longitudinal section is marked perpendicular; cross-section is marked horizontal), thin red arrow: arterioles, black arrows: normal cross striations, thin green arrows: myofibroblasts, thick purple arrow: pericardium, asterix: cavity, thick orange arrow: contraction banding, thick green arrow: atrophic CMs, thick blue arrow: fibrosis. Dotted line surrounds altered areas. Endocardium (thick blue arrow), CMs (thick orange arrow, cross section horizontal, longitudinal perpendicular), Enlarged cell diameter (black line), Cavity (Asterix), Interstitial tissue (blue arrow), Fibrotic area (Black arrow), Myofibroblasts (black arrowheads), Arterioles (red arrowhead).

Anatomically, atria consist of the venous portion, the appendage and the vestibule that leads to the atrioventricular valves. Here we analyzed appendage tissue that is characterized by an array of pectinate muscles^30^. In cross-sections, pectinate muscles display as myocardial islands of different size that are surrounded by endocardial connective tissue (Fig. 1a). In contrast to the atrium, tissue obtained by septal subvalvular left ventricular myectomy presents as a thick layer of relatively uniform myocardium without any crypts^19,31^ (Fig. 1b). Per cross-section, atria show a lower proportion of myocardial tissue and a greater proportion of endocardium and interstitial cells as compared to the ventricle.

**Figure 2:**
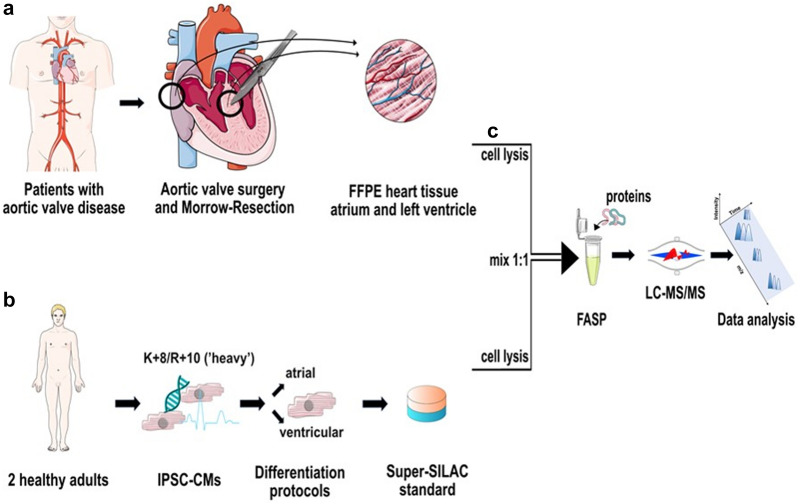
Experimental workflow. (**a**) Atrial and left ventricular samples were taken during aortic valve replacement and Morrow resection. (**b**) iPSC-atrial and ventricular cardiomyocytes were labeled with heavy amino acids and the isolated proteins of three separated replicates of differentiation of iPSCs into atrial and ventricular cardiomyocytes were mixed to establish the protein quantification standard. (**c**) After cell lysis, tissue samples and quantification standard were mixed 1:1 and underwent filter aided sample preparation, followed by high-resolution mass spectrometric analysis. Graphical artwork for this figure was modified from versions provided by Servier Medical Art, licensed under a Creative Common Attribution 3.0 Generic License (www. smart.servier.com).

In healthy ventricular myocardium most of the area is occupied by CMs of normal size (cell diameter < 20 µm), faint cross striations and large nuclei with one or two nucleoli and a delicate interstitium containing fibroblasts and capillaries lined by endothelial cells (Supplementary Fig. 1 and Buetow^31^, Fig. 10.8 C + D and 10.10 C + D, and Basso^32^, Fig. 2e). In contrast, our samples show clear hypertrophic signs, such as enlarged (cell diameter > 20 µm) and tightly packed CMs with partially repressed interstitial area (Fig. 1b) and frequently cellular disarray^33^. Severity of cardiac decompensation is expressed in differed extents of perivascular, interstitial and/or replacement fibrosis (Fig. 1e–g) and areas with pronounced contraction banding CMs (Fig. 1c) and/or atrophic CMs^33,34^ (Fig. 1d). These disease related changes were visible to different extents throughout all specimens.

### Proteomic profiling of FFPE tissue and assessment of SILAC quantification standard

To perform quantitative proteomic profiling of myocardial FFPE tissue using high resolution mass spectrometry, we established the experimental workflow outlined in Fig. 2. Human heart tissue was obtained and immediately formalin-fixed (Fig. 2a). To ensure optimal coverage of the heart proteome, our protein quantification standard consisted of equal amounts of atrial and ventricular iPSC-CMs obtained from two independent iPSC-lines^35^. As iPSC-CMs only poorly divide upon completed differentiation, labeling had to be accomplished by protein turnover. To achieve a labeling efficiency of 98.5%, iPSC-CMs were cultured in SILAC medium for at least 45 days (Fig. 2b) as established previously^16^. Tissue protein was mixed in equimolar amounts with the quantification standard to enable relative quantification of protein abundance. After FASP-based protein extraction, samples were digested with trypsin and resulting peptides were analyzed on a hybrid quadrupole-orbitrap mass spectrometer (Fig. 2c).

**Figure 3:**
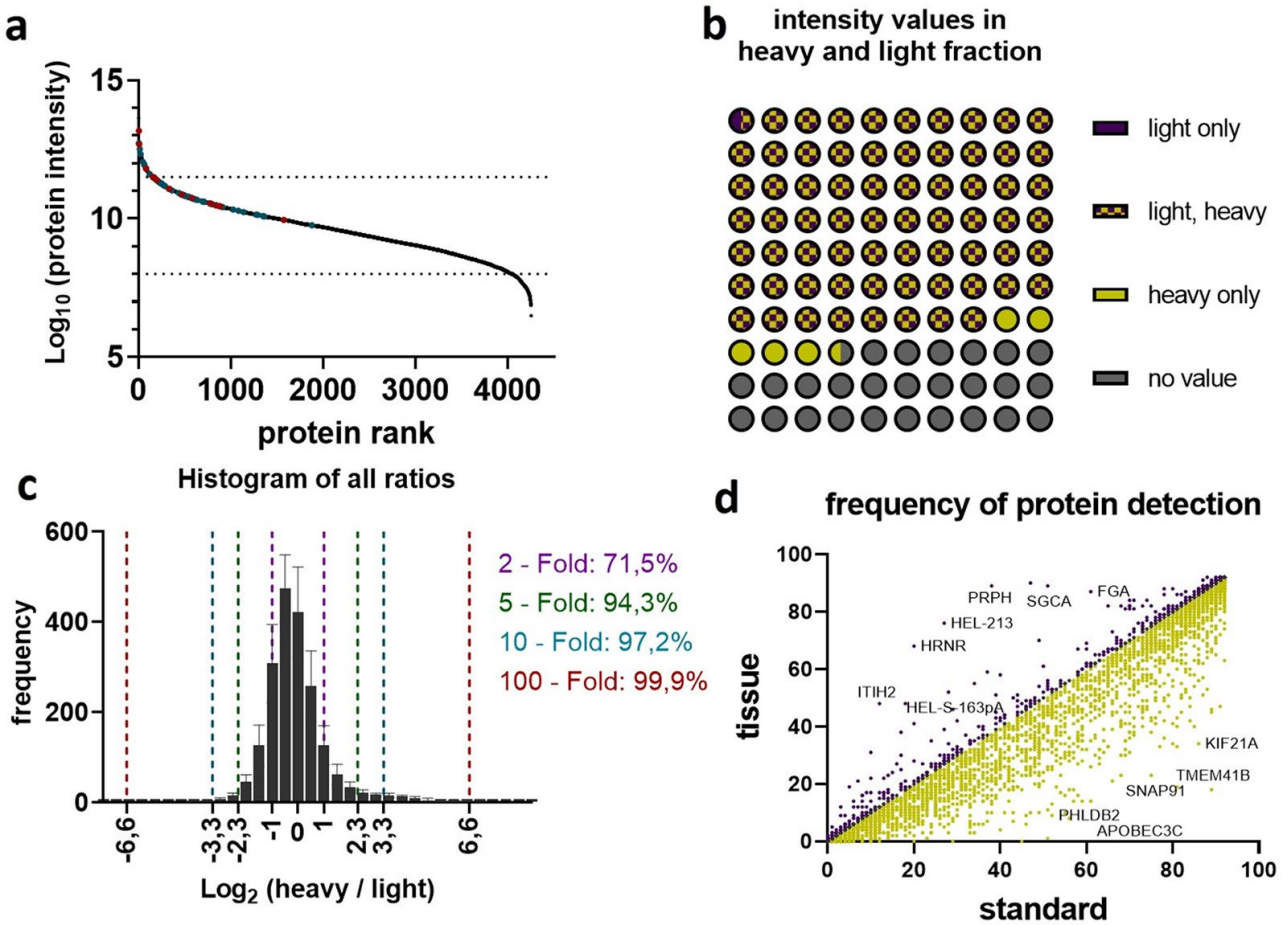
Evaluation of proteomic data and assessment of SILAC standard. (**a**) Ranked abundance plot of all 4,268 proteins detected in the study. Proteins are ranked by their mean log10 MS1 intensities across all experiments, indicating close to 5 order of magnitude linear abundance range. (**b**) Visualization of protein detection completeness. Protein quantitation events are grouped by their percentages of quantifiable values in either SILAC standard (yellow), tissue (purple) or both (checkered) SILAC channels. Protein detections without any quantifiable values are displayed in grey. Less than 1% of protein quantitation events (purple) are not covered by the used SILAC standard. (**c**) Histogram showing the normalized log2 ratio distribution of ratiometric SILAC quantitation values. Values show a normal distribution. (**d**) Frequency of protein detections in the standard versus FFPE tissue with select proteins detected predominantly in tissue or standard.

Protein expression profiles of 46 specimen were analyzed in duplicates. Signal intensities spanned five orders of magnitude, which reflects the identification of proteins with low and high abundance (Fig. 3a). In SILAC approaches, quantification is enabled when a sequence is detected in both fractions, the heavy (standard) and the light (tissue). A good SILAC standard should therefore cover all proteins of the tissue of interest. In 64% of the measures throughout the dataset (provided in Supplementary Table 1) signals were present in both fractions and returned a valid ratio value. In 26.47% signals were missing in both fractions, in 5.57% a sequence was absent in the tissue fraction and in only 0.31% a sequence was absent in the SILAC standard. This indicates a good coverage of heart tissue protein by our SILAC standard (Fig. 3b). Another quality control is the ratio of protein abundance between SILAC standard and tissue protein. More than 90% of all proteins should have a ratio below 5^15^. In our analysis 71.5% of all ratios were within a twofold range, 94.3% within a fivefold range, 97.2% within a tenfold range and 99.9% within a 100-fold range, indicating a good quality of our standard (Fig. 3c). To assess the global difference between SILAC standard, hence iPSC- derived CMs and myocardial tissue, we were also interested in the proteins that were severely more often measured in one or the other fraction. A list of this difference in detection frequency per protein is provided in Supplementary Table 8 and visualized in Fig. 3d. Further MS–MS quality data of proteomic measurements are shown in Supplemental Fig. 2. On the technical level this includes graphical and mean/median assessments of trypsin missed cleavages, peptide length produced, MS/MS counts per peptide, protein molecular weight and protein sequence coverage. All of these are perfectly in line with reported values for most or all protein studies. The only visible limitation here is a slight bias against low molecular weight proteins (5–10 kDa) which is perfectly in line though with the use of bottom-up proteomics approaches in general (less tryptic peptides produced, limited ‘precipitation’ during FASP protocol.

### Differential expression between atria and ventricles

**Figure 4:**
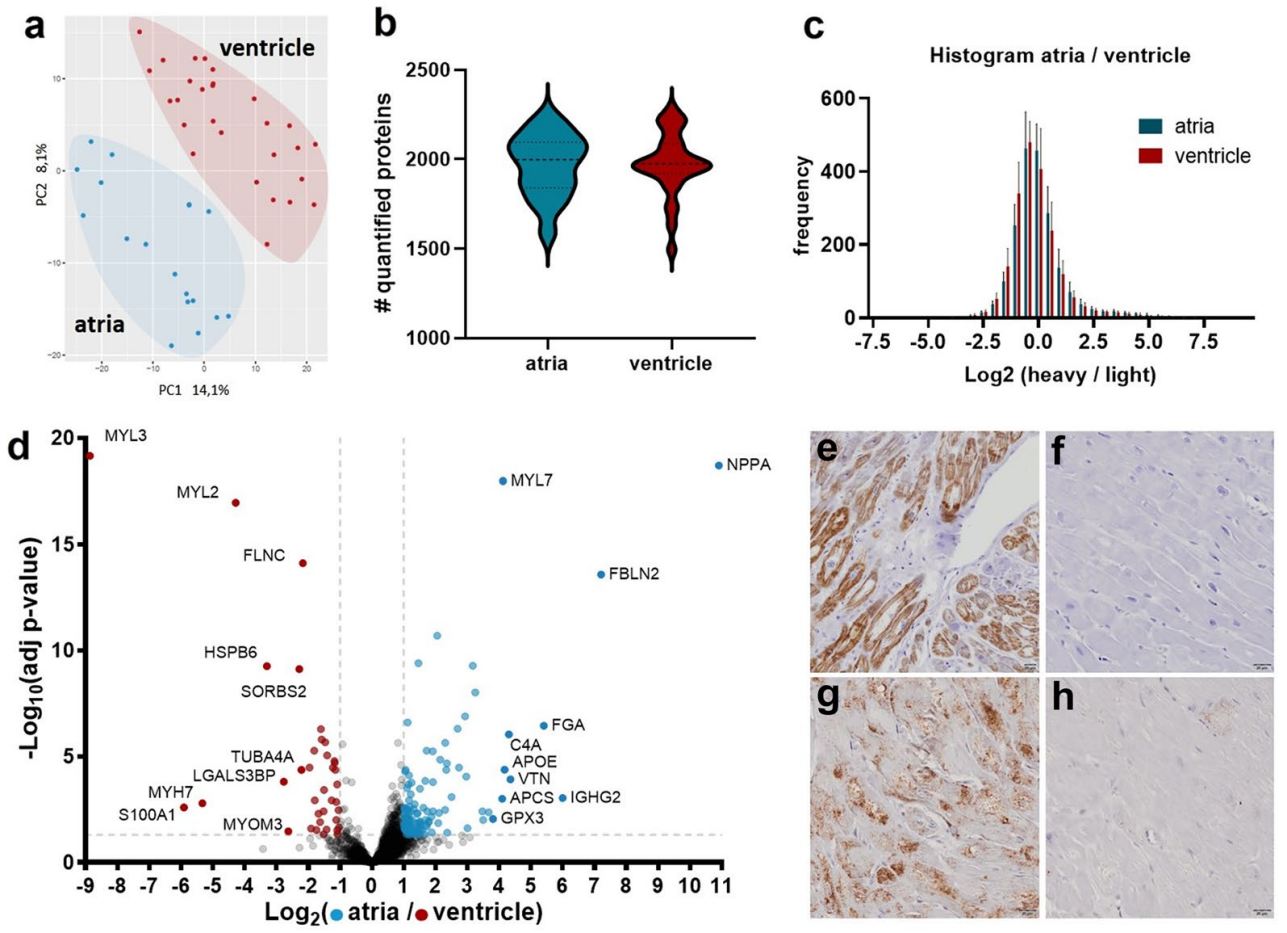
Differentially expressed proteins between atria and ventricles. (**a**) Principal component analysis of atrial (blue) and ventricular (red) protein expression. Shown are the first two principal components. (**b**) Violin plot showing the distribution of the number of quantified proteins per sample, separated into atria and ventricles. While the mean is almost identical between the groups, there is small variation in the range. (**c**) Histogram showing the frequency in which proteins of certain Log2(FC) ratio ranges were measured in atria versus ventricle tissue. (**d**) Volcano plot: adjusted p-values are plotted against the log2 fold change ratios between atria and ventricles. Red (higher expression in ventricles) and blue (higher expression in atria) dots indicate significantly regulated proteins (adj. p < 0.05) with a fold change over 1 or − 1 respectively. (**e**–**h**) Immunhistology, (antibody signal brown, nuclei purple) for MLC2a (**e**, **f**) and NPPA (**g**, **h**) in atrium (**e**, **g**) and ventricle (**f**, **h**).

A principal component analysis separated the specimens precisely into two groups, reflecting distinct differences in the protein expression profiles between atrial and ventricular tissue (Fig. 4a). Sequence alignment returned a total of 4268 protein IDs across all samples. However, a median of 1980 (range 1495–2281) proteins were detected across all samples, with no significant difference in the number of quantified proteins between atria and ventricles (Supplementary Table 1 and Fig. 4b). Also, no significant difference in the normalized log2 ratio distribution of ratiometric SILAC quantification values was detected between atria and ventricles (Fig. 4c).

2,164 proteins had 2/3 or less missing values across all samples and were analyzed for differential expression between atria and ventricles by univariate data analysis using an empirical Bayes method with a linear regression model. Missing values were imputed by using k-nearest-neighbor imputation. These analyses resulted in 293 differential proteins, with 54 upregulated in ventricles and 239 upregulated in atria (Fig. 4d and Supplementary Table 2).

**Figure 5:**
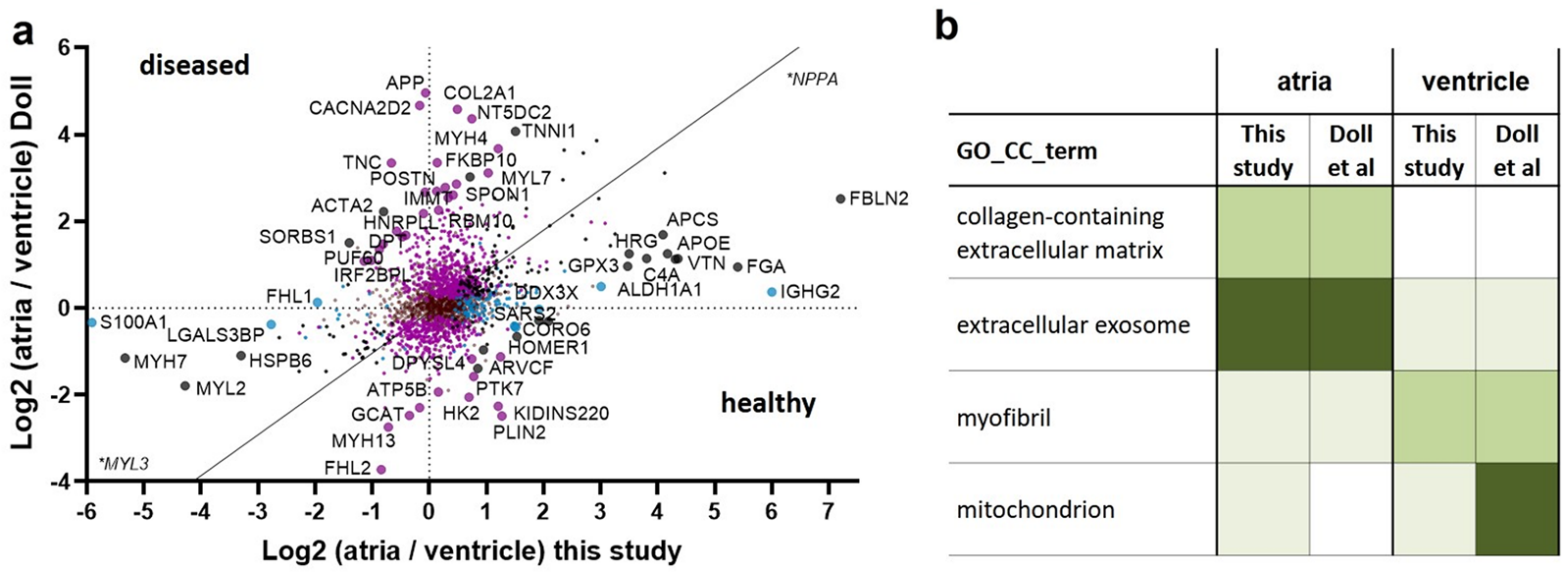
Assessing potentially disease related differences (**a**) Scatterplot comparing the fold change ratios between atria and ventricles for each protein to the fold change ratios published for healthy hearts by Doll et al. Each point represents a protein. Diagonal line: Deming (Model II) linear regression calculated in GraphPad Prism 9 (Y = 0.9427* X—0.09166). Large dots: Proteins with a distance to the regression line of more than 2 in Y direction. Black: proteins significantly regulated (adj. p < 0.05) in both studies. Pink: significantly regulated in Doll et al.^17^ but not in this study. Blue: significantly regulated in this study but not in the study of Doll et al*.* Negative values indicate an upregulation in this study, positive value a regulation towards healthy tissue. (**b**) Overrepresentation analysis of significantly regulated proteins of this and the study of Doll et al*.* White: adj. p value > 0.05, light green: adj. p value > 1 × 10^–15^, green: adj. p value > 1 × 10^–50^, dark green: adj. p value < 1 × 10^–50^.

Providing proof of principle, the known atrium-specific markers PAM and NPPA as well as the atrium-specific myosin isoforms MYL4, MYH6, MYL7 and MLC2a were upregulated in right atria and the ventricle-specific myosin isoforms MYL3 and MYL2 were within the ten most upregulated proteins in left ventricles (Fig. 4d)^36^. Differential abundance of MLC2a and NPPA between atria and ventricles was also demonstrated by immunohistochemistry (Fig. 4e–h). The difference in the total number of upregulated proteins in the atria versus the ventricle is in line with the current literature^16,17,37–39^ and reflects a higher cellular and extracellular diversity of atrial myocardium^40^. Overrepresentation analysis revealed a global upregulation of proteins related to cardiac contraction in the ventricles and an upregulation of proteins related to the ECM and the secretory system in the atria (Fig. 5b). This resembles the histologically visible findings on the distribution of these compartments in atria versus the ventricle (Fig. 1a,b) and is also in line with the current literature^4,17,38,40^.

### Proteomic data comparison between AVS and healthy hearts

To detect protein expression signatures related to aortic valve disease, a comparison to healthy heart tissue was necessary. As our dataset lacked an internal healthy control, we used the published dataset from Doll et al., who had performed a differential expression analysis between atrial (left, right and septum) and ventricular (left, right and septum) proteomes of 3 postmortem healthy human hearts^17^. File 41467_2017_1747_MOESM7, published in the Supplemental material by Doll et al*.*^17^ was used for comparison. This dataset contained 8829 differential proteins and covered 98% of the proteins that we used for differential expression analysis.

We first performed overrepresentation analysis using all significantly regulated atrial and ventricular proteins from the current study and the dataset of Doll et al.^17^. GO-terms such as collagen-containing extracellular matrix, extracellular exosome and myofibril showed an overall comparable chamber distribution between the studies. Markedly, the term “mitochondrion” was highly significant (adj. p-value: 1.5E − 132) in healthy ventricles, while in diseased hearts the term was much less significant (adj. p-value: 4.77E − 05) and even present in the atrium (adj. p-value: 1.07E − 07) (Fig. 5b and Supplementary Table 3). This data indicates a strong disbalance in mitochondrial proteins and can count as a clear sign for mitochondrial impairment in the diseased ventricles. To detect potential disease-related proteins directly, we performed a detailed protein–protein comparison between ours and the dataset of Doll et al. (Supplementary Table 4)^17^.

The log_2_ fold change ratios between atria and ventricles of both studies were plotted and orthogonal linear regression was calculated. All proteins with a Y-distance from the regression line greater than 2 and with a significant fold change in at least one of the studies were defined as potentially disease-relevant (Fig. 5a and Supplementary Table 4).

An enrichment of sarcomeric proteins (MYH7, MYL3, SORBS1, TNNI1, MYH4, MYL2, ACTC1 and MYL7) in diseased ventricular myocardium reflects increased muscle mass due to cardiac hypertrophy^4,33^. Furthermore, we detected several hypertrophy related proteins (S100A1, HDAC2, IRF2BPL, RBM10, FHL1, FHL2, HOMER1, APOE, PLIN2 and FBLN2). Consistent with the pathophysiology in hypertrophy, we identified high abundant blood supply related proteins (HRG, HBB, HBA, FGG, GPX3, C4A and FGA). These findings underline the increasing need of nutrition and the reduced blood supply that often occurs due to the enhanced spatial demands of hypertrophic CMs that compress arterioles^4,5^.

Cardiac fibrosis and enhanced deposition of ECM are signs for decompensation in hypertrophy^4,32,34^. Indicating a high incidence of fibrosis in diseased tissue, we found fibrosis related proteins (APP, FKBP10, HSPB6 and APCS). We also found ECM related proteins (COL2A1, TNC, FKBP10, LTBP1, LGALS3BP, DPT and SPON1) upregulated in diseased tissue, while PTK7 and VTN were more abundant in healthy tissue.

Mitochondrial impairment is a hallmark of decompensation in hypertrophic hearts^41^. Indicating mitochondrial impairment, we found downregulated mitochondrial proteins (SARS2, DDX2X, DUT CLICS, ATP5B, ALDH1A1, DPYSL4 and HK2). However, the disease related mitochondrial proteins IMMT^42^ and PDK3^43^ that are known to be upregulated in hypertrophic but not healthy mitochondria were enriched in diseased tissue.

All the above-mentioned proteins can be related to cardiac hypertrophy and/or cardiac decompensation, and therefore validate the conducted experimental approach. Further potentially disease-relevant protein candidates without a known functional relation to hypertrophy, fibrosis or decompensated heart disease were SLTM, HNRNPLL, FIP1L1, RNMT, KRT18 and PUF60 and the downregulated NUDT4, GCAT, KIDINS220 and ARVCF.

## Discussion

Surgically excised human heart tissue is routinely stored in Formalin and embedded in Paraffin (FFPE) for different purposes. Biobanks worldwide store huge amounts of FFPE tissue. This biobank material is of great value and novel techniques enable the use of high-resolution mass spectrometry. However, quantification of FFPE heart tissue has been limited due to methodical issues. Here we established an experimental workflow that combines filter-aided sample preparation with an iPSC-derived heart-specific and SILAC-labeled protein quantification standard for high-resolution mass spectrometry to identify disease-related proteins in FFPE heart tissue. The substantial advantage of using a protein quantification standard, beside the ability to add samples to the analysis that were measured temporally apart, is the high accuracy and reliance of the mass spectrometric analysis^15^.

In our investigated cohort, all patients showed severe AVS and had developed a septal cardiac hypertrophy with an additional need of Morrow resection^18,19^. When such patients develop the symptoms of heart failure, they are in urgent need for surgery to prevent sudden cardiac death^9,44^. A deeper understanding of the molecular and cellular pathophysiology of AVS induced heart failure could improve patient risk stratification, disease monitoring and clinical management of affected patients^3^.

Using our established workflow, we were able to quantify 2164 proteins from 29 left ventricular and 17 right atrial human FFPE tissue. 239 of those proteins were upregulated in atrial and 54 proteins in ventricular myocardium. The higher number of upregulated proteins in the atria correlates with the current literature^17,38,40^ and reflects the higher cellular and extracellular diversity of atrial myocardium^40^. While in the ventricle, myocardium is marked by tightly packed CMs with rare interstitial space, atria display a larger interstitial compartment with diverse cell types and ECM proteins and the trabecular structure causes a higher content of endocardium^31^.

Patients with AVS develop left ventricular hypertrophy as an adaptive remodeling response due to the increased cardiac workload^9^. While CMs enlarge through protein synthesis and sarcomeric re-organization, the overall cardiac muscle mass increases^4^. Cardiac hypertrophy becomes maladaptive when cell death, fibrosis, dysregulation of Ca^2+^-handling proteins, mitochondrial dysfunction, metabolic reprogramming, reactivation of fetal gene expression, impaired protein, and mitochondrial quality control, altered sarcomere structure, and insufficient angiogenesis occurs^4^. The grade of preoperative myocardial fibrosis has emerged as the strongest independent predictor of mortality in AVS^6,8^.

In our histological staining key features of cardiac hypertrophy were displayed, such as enlarged and tightly packed CMs, as well as signs for decompensation, such as fibrotic lesions and altered sarcomeres in degenerating CMs^33^.

Our proteomic dataset reflects the key features of cardiac hypertrophy and cardiac decompensation. Enhanced muscle mass was detected in diseased tissue. Overrepresentation analysis and protein–protein comparison between healthy and diseased tissue revealed a mitochondrial impairment in diseased tissue. Furthermore, our data suggests a reduction in blood supply^4^ that is known to cause ischemia^5^ and a high abundance of proteins related to fibrosis and the ECM.

Literature search and comparison of each single potentially disease relevant protein strengthens the reliability of our dataset by confirming their relation to the above-mentioned pathophysiologic changes in AVS. Chances are high, that proteins listed in our dataset but not yet connected to the disease might also play a role in the development of decompensated AVS.

In summary, our study provides a reproducible blueprint for quantitative proteomic analysis of FFPE human heart tissue samples. Furthermore, it could serve as a comprehensive proteomic resource for human atrial and ventricular tissue of patients with severe AVS and it shows a well selected list of potentially disease-related proteins, worth to be further investigated for a better understanding of the pathophysiology in the myocardium of AVS and the development of potential therapeutic and diagnostic strategies.

## Limitations

The major limitation of our study was the lack of an internal healthy control. To overcome this limitation and gain insight into the proteomic changes induced by AVS, we calculated the log_2_ fold change ratios between atria and ventricles of our diseased hearts and compared those to published ratios from three healthy hearts^15^. While we used tissue from the right atrium obtained during the cannulation procedure and subvalvular left ventricular myocardium from the septum, Doll et al*.* combined tissue from the left and right atrium and the atrial septum and tissue from the left and right ventricle and the ventricular septum respectively. This is indeed a source of error. However, principal component analysis of the 16 heart regions that were analyzed in Doll et al.’s publication indicated a much higher difference between atrial and ventricular regions together, than between the right, left and septal regions. We therefore assume a subordinate role for this error.

Another potential error is the difference between the protocols that were used for protein isolation and mass spectrometry. Our tissue was immediately fixed in formalin, and we used an internal SILAC standard, resulting in a ratio between heavy labeled proteins and FFPE tissue proteins. Doll et al.’s tissue was collected less than 72 h postmortem during an autopsy after a court order and proteins were measured directly without an internal SILAC standard. This resulted in 2164 quantifies proteins with 239 enriched in the atria and 54 enriched in the ventricles using our protocol, and in 11,236 quantified proteins with 1220 enriched in the atrium and 409 enriched in the ventricle. Doll et al.’s dataset shows a much higher resolution compared to ours and indeed it covers almost all of the proteins that we worked with, making it extremely suitable as a reference. However, a direct comparison between the proteins that were enriched in our ventricles to healthy ventricular protein would be misleading. Here we chose to compare the ratios of the atrium and the ventricle, which allows to neglect most procedural differences as those cut out. However, what our approach cannot correct for is selective recovery of individual parts of the protein population, as observed in kidney by Amarnani et al.^45^. In overrepresentation analysis, we used the complete lists of proteins, while for protein-to-protein comparison we only analyzed the proteins present in both studies.

Another limitation was that atrial fibrillation, arterial hypertension and ischemic heart disease of many patients might lead to confounding results, but statistical analysis of the proteome expression profiles of non-ischemic versus ischemic diseased hearts (Supplementary Tables 6 and 7) and with or without atrial fibrillation (Supplementary Table 5) did not provide any significant differences in protein abundance. Genetic hypertrophic obstructive cardiomyopathy, rheumatic disease and low flow aortic valve stenosis were excluded from the analysis. The normalization against atrial tissue hereby reduced potential bias from patient cohort specific differences such as age, sex and concomitant conditions (e.g., diabetes, medication).

## Supplementary Information

Supplementary Figure 1.

Supplementary Figure 2.

Supplementary Information.

Supplementary Table 1.

Supplementary Table 2.

Supplementary Table 3.

Supplementary Table 4.

Supplementary Table 5.

Supplementary Table 6.

Supplementary Table 7.

Supplementary Table 8.

## Abbreviations

ABC: Ammonium bicarbonate
AVS: Aortic valve stenosis
CABG: Coronary artery Bypass graft
CMs: Cardiomyocytes
DCA: Deoxycholic acid
ECM: Extracellular matrix
FASP: Filter-aided-sample preparation
FFPE: Formalin-fixed and paraffin-embedded
GSEA: Gene set enrichment analyses
GO: Gene ontology
GO-CC: Gene ontology for cellular components
iPSC: Induced pluripotent stem cells
iPSC-CMs: Induced pluripotent stem cell derived cardiomyocytes
MS: Mass spectrometry
NYHA: New York Heart Association
SILAC: Stable isotope labeling by/with amino acids in cell culture
TFA: Trifluoroacetic acid
