# Supplementary material for: Proteomic mapping of atrial and ventricular heart tissue in patients with aortic valve stenosis
Source: Sci Rep. 2021 Dec 22;11:24389. doi: 10.1038/s41598-021-03907-3 (PMC8695579; doi:10.1038/s41598-021-03907-3)
Supplement: Supplementary file 1 — Supplementary Figure 1. [file 41598_2021_3907_MOESM1_ESM.pdf]

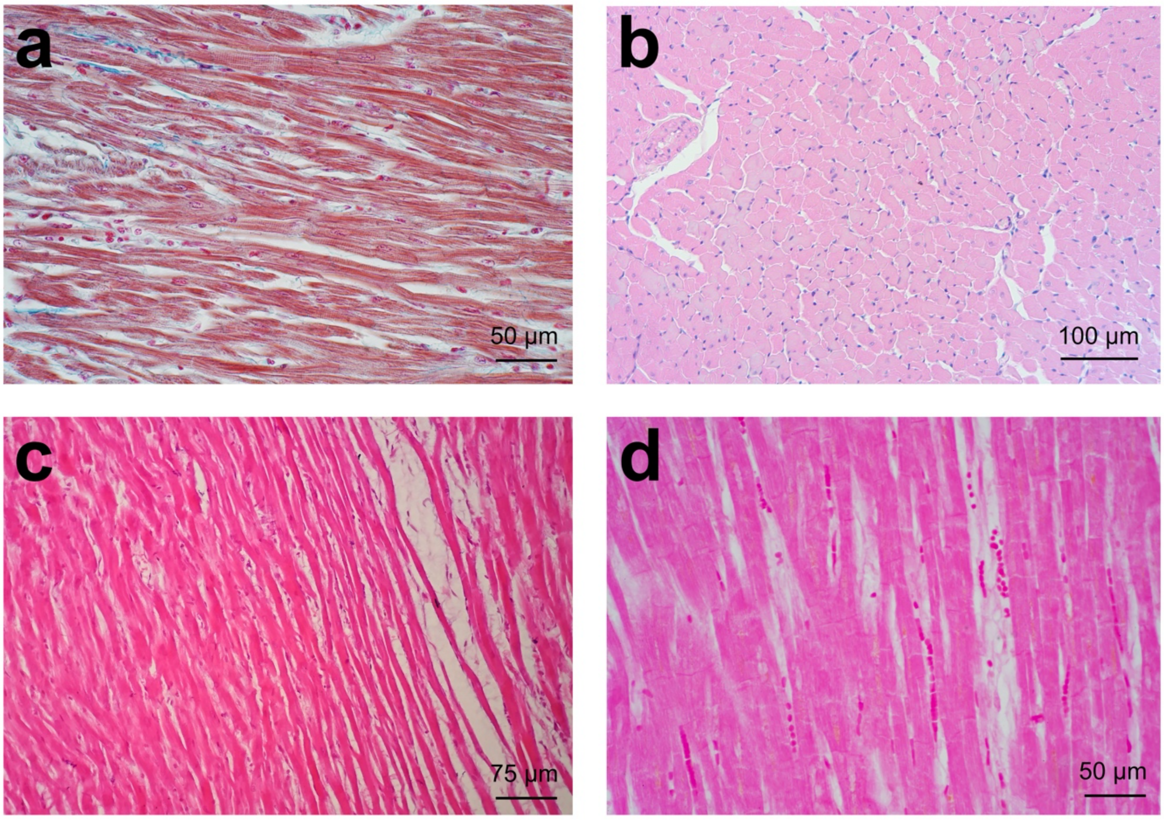

Supplementary Figure 1. Histology of healthy human heart:
In healthy ventricular myocardium most of the area is occupied by cardiomyocytes of normal size (cell diameter <20µm), faint cross striations and large nuclei with one or two nucleoli and a delicate interstitium containing fibroblasts and capillaries lined by endothelial cells. The pictures shown were obtained from www.shutterstock.com and examined and validated by pathologists.
