# Supplementary material for: Proteomic mapping of atrial and ventricular heart tissue in patients with aortic valve stenosis
Source: Sci Rep. 2021 Dec 22;11:24389. doi: 10.1038/s41598-021-03907-3 (PMC8695579; doi:10.1038/s41598-021-03907-3)
Supplement: Supplementary file 2 — Supplementary Figure 2. [file 41598_2021_3907_MOESM2_ESM.pdf]

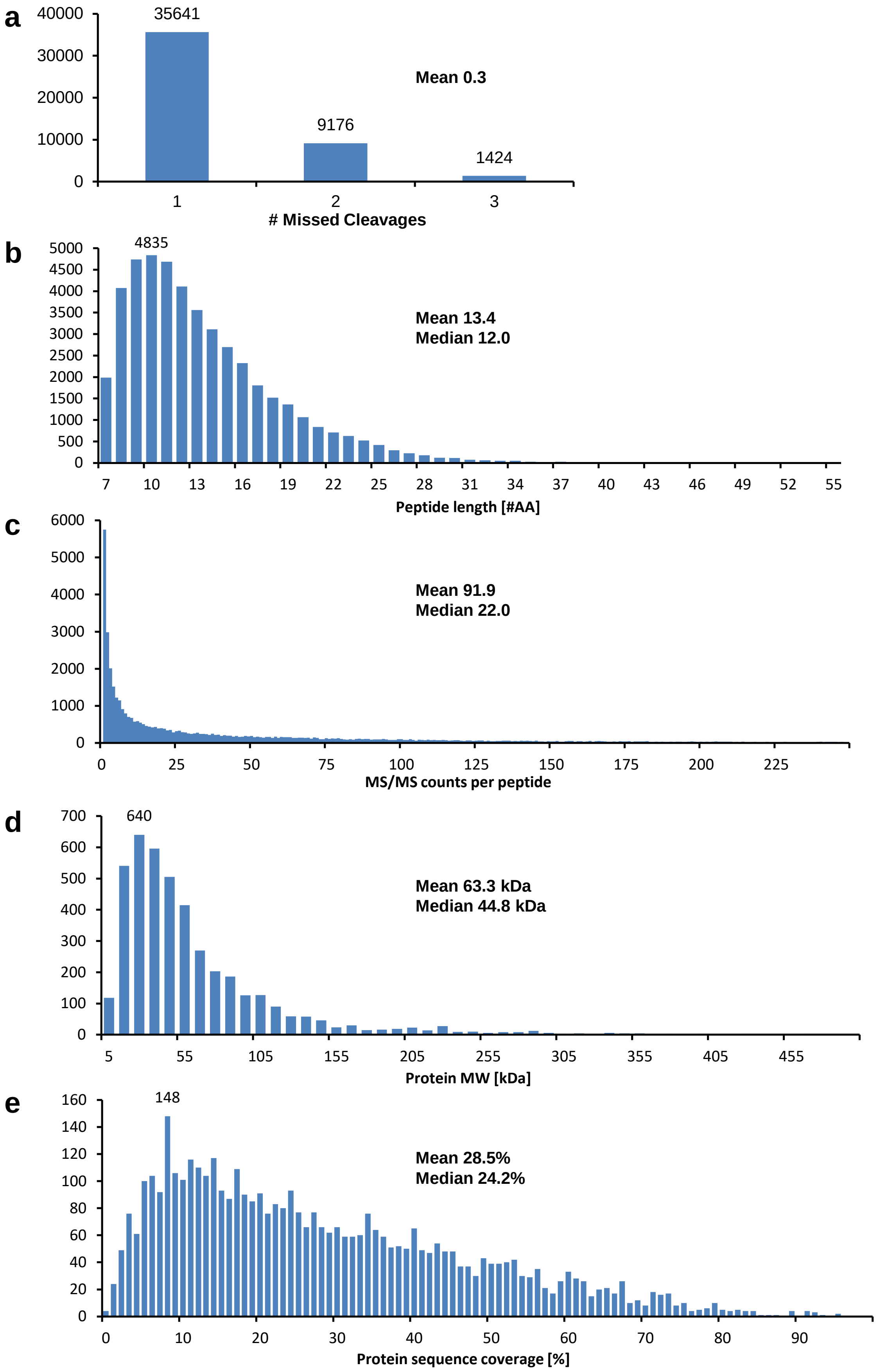
a
40000
30000
20000
10000
0
35641
9176
1424
Mean 0.3
1
2
3
Missed Cleavages
b
5000
4500
4000
3500
3000
2500
2000
1500
1000
500
0
4835
Mean 13.4
Median 12.0
7
10
13
16
19
22
25
28
31
34
37
40
43
46
49
52
55
Peptide length [#AA]
c
6000
5000
4000
3000
2000
1000
0
Mean 91.9
Median 22.0
0
25
50
75
100
125
150
175
200
225
MS/MS counts per peptide
d
700
600
500
400
300
200
100
0
640
Mean 63.3 kDa
Median 44.8 kDa
5
55
105
155
205
255
305
355
405
455
Protein MW [kDa]
e
160
140
120
100
80
60
40
20
0
148
Mean 28.5%
Median 24.2%
0
10
20
30
40
50
60
70
80
90
Protein sequence coverage [%]

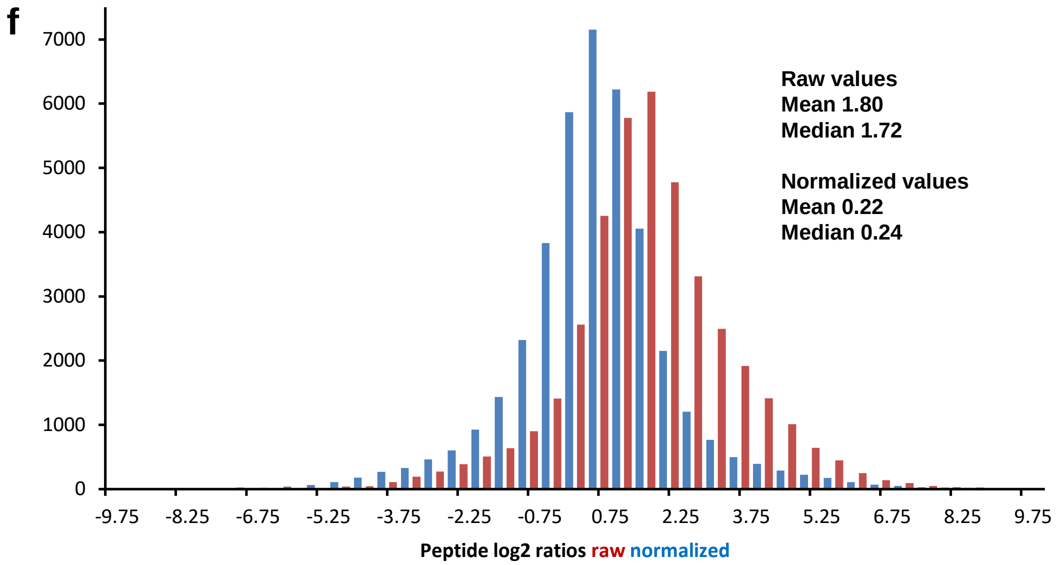
f
7000
6000
5000
4000
3000
2000
1000
0
-9.75
-8.25
-6.75
-5.25
-3.75
-2.25
-0.75
0.75
2.25
3.75
5.25
6.75
8.25
9.75
Raw values
Mean 1.80
Median 1.72
Normalized values
Mean 0.22
Median 0.24
Peptide log2 ratios raw normalized

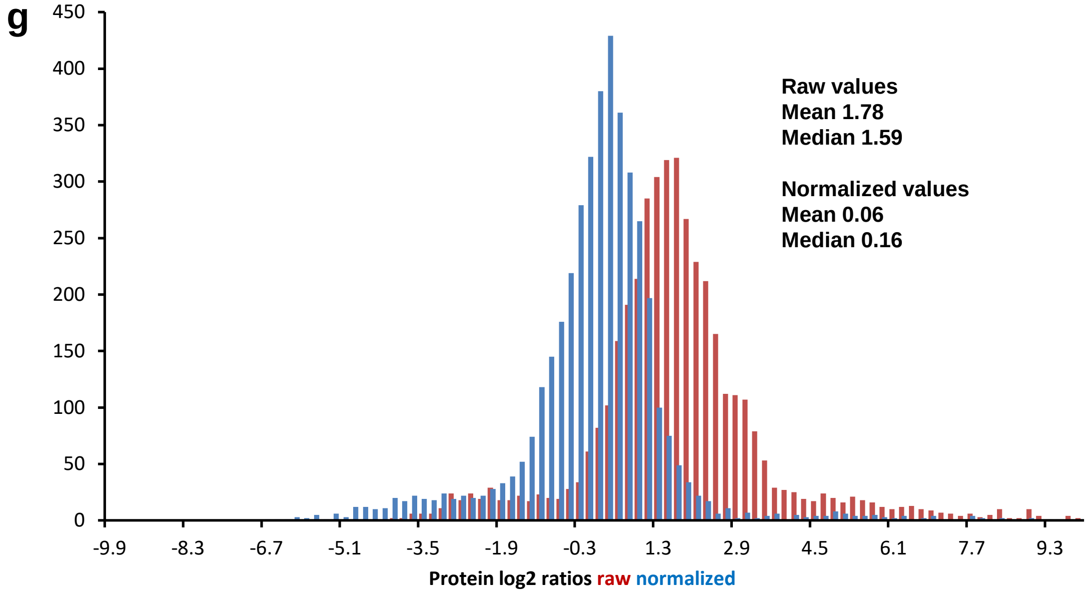
g
450
400
350
300
250
200
150
100
50
0
-9.9
-8.3
-6.7
-5.1
-3.5
-1.9
-0.3
1.3
2.9
4.5
6.1
7.7
9.3
Raw values
Mean 1.78
Median 1.59
Normalized values
Mean 0.06
Median 0.16
Protein log2 ratios raw normalized

Supplementary Figure 2: Histograms of key quality control parameters for mass spectrometric proteome analysis (peptides n=42,641, proteins n=4,268, all before filtering). Mean and median values are given in the respective panels. (a) distribution of missed cleavages in the identified peptides, (b) distribution of peptide lengths, (c) distribution of MS/MS counts per peptide, (d) binned molecular weight distribution of proteins (bin size 10 kDa), (e) sequence coverage distribution of proteins. Histograms of (f) peptide and (bg) protein log 2 SILAC ratio distributions before (red) and after (blue) normalization (peptides n=42,641, proteins n=4,268, all before filtering). Mean and median values are given in the respective panels. Distribution shifts indicate a slight over-spiking of the heavy-labeled SILAC standard, which however is desirable to obtain near-complete coverage of endogenous peptide/proteins by the standard.
