# Supplementary material for: Proteomic mapping of atrial and ventricular heart tissue in patients with aortic valve stenosis
Source: Sci Rep. 2021 Dec 22;11:24389. doi: 10.1038/s41598-021-03907-3 (PMC8695579; doi:10.1038/s41598-021-03907-3)
Supplement: Supplementary file 3 — Supplementary Information. [file 41598_2021_3907_MOESM3_ESM.pdf]

1. FASP protocol for the proteomic analysis of formalin-fixed tissue samples

Material

1. Xylene
2. Absolute ethanol
3. Lysis Buffer: 4% SDS, 0.2% DCA, 50 mM TCEP, 100 mM ABC, pH 8
4. 660 nM Kit + Ionic Detergent Compatibility Reagent (Thermo Scientific)
5. Exchange Buffer: 8 M urea, 0.2% DCA, 100 mM ABC, pH 8
6. Alkylation Buffer: 8 M urea, 50 mM IAA, and 100 mM ABC, pH 8
7. Digestion Buffer: 0.2% DCA, 50 mM ABC, pH 8
8. Peptide Recovery Buffer: 50 mM ABC, pH 8
9. Ethyl acetate
10. Trifluoroacetic acid (TFA)
11. Methanol 50%
12. Microcon® UF unit YM-30
13. Eppendorf LoBind® tube, 1,5 mL

Day 1

1. Cut ~5 rolls of 10 micro meter of FFPE tissue using macrodissection
2. Add 1ml of xylene to the FFPE tissue slices, vortex and incubate it with gentle agitation (600rpm) at room temperature for 5 min. Centrifuge at max speed for 5 min at room temperature.
3. Remove the solution, add 1 mL of xylene, vortex and incubate as in (2). Centrifuge at max speed for 5 min.
4. Remove the solution and repeat steps 2 and 3 in each using 1 mL of absolute ethanol. Centrifuge at max speed for 5 min.
5. Remove ethanol and vacuum-dry the sample at 45°C.
6. Mix the dried tissue with 250 - 500 µl lysis buffer (depending on the amount of tissue) and homogenize using a disperser.
7. Mix at 600 rpm in a thermo-mixer for 60 minutes at 95°C.
8. Sonicate the suspension for 3 min.
9. Centrifuge at max speed for 15 minutes.
10. Mix equal amounts of clarified lysate and super-silac-standard (each 25 µg protein) with 200 µL of Exchange Buffer in the filter unit and centrifuge at 14,000 rcf for 10 min at 20°C
11. Add 200 µL Exchange Buffer and centrifuge at 14,000 g for 10 minutes at 20°C. Discard filtrate. Repeat this step twice.

12. Add 100 µL Alkylation Solution, and mix at 300 rpm in a thermo-mixer for 60 min at 37°C protected from light.
13. Centrifuge at 14,000 g for 10 minutes at 20°C. Discard filtrate.
14. Add 200 µL Exchange Buffer and centrifuge at 14,000 g for 10 minutes. Discard filtrate.
15. Add 200 µL Digestion Buffer and centrifuge at 14,000 g for 10 minutes. Discard filtrate. Repeat this step twice.
16. Transfer the filter unit to a new collection tube.
17. Add 90 µL eFASP Digestion Buffer to each filter unit.
18. Add Trypsin to the filter unit (trypsin:protein 1:50 w:w -> 1µg Trypsin for 50µg).
19. Mix at low speed (300rpm) in a thermo-mixer for overnight at 37°C.

Day 2

1. Centrifuge at 14,000 g for 10 minutes at 20°C.
2. Add 50 µL of Peptide Recovery Buffer to each filter unit and centrifuge at 14,000 g for 10 minutes at 20°C. Repeat this step once.
3. Add 200 µL of ethyl acetate to the peptide-containing filtrate and transfer to a 1,5mL Eppendorf LoBind® tube.
4. Add 2.5 µL TFA and quickly vortex. White thread-like precipitate may be visible for large quantities of peptides.
5. Add 200µl ethyl acetate.
6. Sonicate for 10 seconds and centrifuge at 18,000 rcf for 10 minutes at 20°C.
7. Carefully remove and discard as much of the upper organic layer.
8. Repeat steps 6 through 8 twice.
9. Place the uncovered sample tube in a thermo-mixer at 60°C, in a fume hood, for 5 minutes to remove residual ethyl acetate.
10. Remove residual organic and volatile salts by vacuum drying in a SpeedVac at 45°C.
11. Thoroughly resuspend in 50 µL 50% methanol and vacuum-dry 2 times.
12. Thoroughly resuspend in 60 µL 50% methanol, vortex for 10s, incubate in a thermomixer at 37°C for 10min at 600rpm and vortex again for 10s.
13. Vacuum-dry the sample and store at -80°C until mass spectrometric analysis.
